# Imaging the WHO 2021 Brain Tumor Classification: Fully Automated Analysis of Imaging Features of Newly Diagnosed Gliomas

**DOI:** 10.3390/cancers15082355

**Published:** 2023-04-18

**Authors:** Michael Griessmair, Claire Delbridge, Julian Ziegenfeuter, Denise Bernhardt, Jens Gempt, Friederike Schmidt-Graf, Olivia Kertels, Marie Thomas, Hanno S. Meyer, Claus Zimmer, Bernhard Meyer, Stephanie E. Combs, Igor Yakushev, Benedikt Wiestler, Marie-Christin Metz

**Affiliations:** 1 Department of Neuroradiology, Klinikum Rechts der Isar, TU Munich, 81675 Munich, Germany; 2 Department of Pathology, Klinikum Rechts der Isar, TU Munich, 81675 Munich, Germany; 3 Department of Radiation Oncology, Klinikum Rechts der Isar, TU Munich, 81675 Munich, Germany; 4 Department of Neurosurgery, Klinikum Rechts der Isar, TU Munich, 81675 Munich, Germany; 5 Department of Neurosurgery, University Medical Center Hamburg-Eppendorf, 20246 Hamburg, Germany; 6 Department of Neurology, Klinikum Rechts der Isar, TU Munich, 81675 Munich, Germany; 7 Department of Nuclear Medicine, Klinikum Rechts der Isar, TU Munich, 81675 Munich, Germany; 8 TranslaTUM, TU Munich, 81675 Munich, Germany

**Keywords:** glioma, WHO CNS 2021, methylation analysis

## Abstract

**Simple Summary:**

With the release of the fifth WHO classification for CNS tumors in 2021, biology-based tumor classification is further advanced with the addition of molecular characteristics into diagnosis. This both poses challenges as well as opens up new opportunities for radiological diagnosis, which was long based on the correspondence of imaging features and histological criteria. In this work, using advanced imaging and AI-based image processing on newly-diagnosed adult glioma patients (n = 226) with extensive molecular characterization, significant differences in biological MR imaging metrics among molecularly defined glioma subgroups were demonstrated. In particular, diffuse glioma (*IDH* wild type) with molecular characteristics of glioblastoma (now recognized as glioblastoma, WHO CNS grade 4) showed higher perfusion as well as increased cell density compared to “classical” glioblastoma (*IDH* wild type), WHO CNS grade 4, and astrocytoma (*IDH* mutant, 1p/19q non-codeleted), WHO CNS grade 4. Our results add relevantly to the emerging picture that fine tumor grading is possible in part by visualization of tumor biology with advanced MRI.

**Abstract:**

Background: The fifth version of the World Health Organization (WHO) classification of tumors of the central nervous system (CNS) in 2021 brought substantial changes. Driven by the enhanced implementation of molecular characterization, some diagnoses were adapted while others were newly introduced. How these changes are reflected in imaging features remains scarcely investigated. Materials and Methods: We retrospectively analyzed 226 treatment-naive primary brain tumor patients from our institution who received extensive molecular characterization by epigenome-wide methylation microarray and were diagnosed according to the 2021 WHO brain tumor classification. From multimodal preoperative 3T MRI scans, we extracted imaging metrics via a fully automated, AI-based image segmentation and processing pipeline. Subsequently, we examined differences in imaging features between the three main glioma entities (glioblastoma, astrocytoma, and oligodendroglioma) and particularly investigated new entities such as astrocytoma, WHO grade 4. Results: Our results confirm prior studies that found significantly higher median CBV (*p* = 0.00003, ANOVA) and lower median ADC in contrast-enhancing areas of glioblastomas, compared to astrocytomas and oligodendrogliomas (*p* = 0.41333, ANOVA). Interestingly, molecularly defined glioblastoma, which usually does not contain contrast-enhancing areas, also shows significantly higher CBV values in the non-enhancing tumor than common glioblastoma and astrocytoma grade 4 (*p* = 0.01309, ANOVA). Conclusions: This work provides extensive insights into the imaging features of gliomas in light of the new 2021 WHO CNS tumor classification. Advanced imaging shows promise in visualizing tumor biology and improving the diagnosis of brain tumor patients.

## 1. Introduction

For over 40 years, the WHO (World Health Organization) has published a classification for CNS (central nervous system) tumors, serving as a normalized standard in both clinical practice and neuro-oncology research. With the publication of the fifth edition of the WHO classification for CNS tumors in November 2021, a more specific classification and the introduction of new tumor entities were launched due to the increasing focus on molecular characteristics [1]. However, as histopathological and immunohistochemical methods continue to play a crucial role, the combination of molecular and histological analysis in the new WHO classification should be understood as an improved tool for an integrated diagnosis. As a main novelty and emphasizing the importance of molecular characterization, a homozygous deletion of *CDKN2A/B* is sufficient to graduate *IDH* mutant astrocytomas without 1p/19q codeletion as WHO CNS grade 4 or oligodendrogliomas with 1p/19q codeletion as WHO CNS grade 3, even without the histological hallmarks of a high-grade glioma [2]. These innovations pose new challenges to radiological tumor grading, which in the past was based primarily on the correspondence of imaging and histological features. Many studies have already shown correlations between imaging biomarkers such as ADC (apparent diffusion coefficient), contrast uptake, and rCBV (relative cerebral blood volume) and tumor types and grading. These data, except for Yang et al. [3], refer to the older WHO classifications [4,5,6,7,8]. Although diagnosis can now usually be made accurately using EPIC array methylation analysis (Illumina Inc., San Diego, CA, USA) or single marker testing, these molecular methods provide only a temporal and regional snapshot of the tumor, confined by the extent of the provided histopathological sample and the date of resection, and do not necessarily capture the aspect of intratumoral heterogeneity and evolution [9]. As a synergistic tool, a profound radiological tumor assessment could provide information at different time points. In addition, in contrast to a selective biopsy, the entire tumor volume and consequently intratumoral heterogeneity would be included [10,11]. These aspects might have an important effect on early diagnosis, better therapy planning, and more precise follow-up. As described above, there are currently few studies that assess correlations between imaging biomarkers and the new tumor entities. However, due to different mutation profiles and, therefore, biological behaviors of the tumors, differences could be expected. Thus, the aim of this work was to identify differences in the imaging phenotype of gliomas as defined by the fifth edition of the WHO classification of tumors of the CNS. A special focus was put on possible differences between astrocytomas of WHO CNS grade 4 (*IDH* mutant), glioblastomas of WHO CNS grade 4 (*IDH* wild type), and diffuse gliomas (*IDH* wild type) with the molecular characteristics of a glioblastoma of WHO CNS grade 4, which now also counts as a glioblastoma (*IDH* wild type).

## 2. Materials and Methods

### 2.1. Patient Selection

This retrospective analysis of a mono-centric prospective observational glioma cohort included newly diagnosed adult glioma patients (n = 226). Inclusion criteria were preoperative MR imaging as well as neuropathological and molecular evaluation, allowing classification according to the new WHO classification 2021, as detailed below [1]. Patients provided written informed consent for inclusion into the prospective glioma registry, which was approved by our local IRB.

### 2.2. Neuropathology and Methylation Analysis

Tissue samples of 226 gliomas were formalin-fixed and paraffin-embedded for standard neuropathological diagnosis. In addition to conventional histomorphological and immunohistochemical examinations, DNA was extracted from the most representative, selected tumor areas, and 850 K methylation analysis (using Illumina EPIC 850 K Methylation Array BeadChip, Illumina Inc., San Diego, CA, USA), including classification by the Brain Tumor Classifier of the DKFZ and Heidelberg University Hospital, was performed as previously described [12,13]. An integrated diagnosis, taking into account the histology, immunohistochemistry, and molecular data, was then determined by two board-certified neuropathologists, according to standards set by the current WHO classification.

### 2.3. MRI Analysis

All available preoperative MR images from a patient were rigidly co-registered into SRI24 atlas space [14] with NiftyReg [15] and skull-stripped using HD-BET [16]. FLAIR, T2w, and T1w images with and without contrast agents served as the basis for a fully automated tumor segmentation into necrosis, contrast-enhancing tumor, and edema/T2-hyperintense tumor areas with the BraTS. Toolkit [17], which implements several state-of-the-art glioma segmentation algorithms as well as fusion. In the case of missing conventional images (mostly T2w), we employed a GAN (generative adversarial network)-based approach for the synthesis of missing modalities [18].

For post-processing the advanced imaging modalities (diffusion and perfusion imaging), we used dipy [19] for calculating diffusivity (ADC) and fractional anisotropy (FA) maps from diffusion-tensor imaging and a leakage-correction algorithm for CBV maps by Arzanforoosh et al. [20]. The latter also requires a mask of the normal-appearing white matter to normalize the resulting CBV, which we generated using ANTs Atropos [21] while excluding tumor areas.

All registrations and postprocessing results were visually verified by M.G. and M.M. Furthermore, to validate the accuracy of the automatic segmentations for our data, a subcohort of n = 22 glioma patients was randomly chosen and manually segmented into necrosis, contrast-enhancing tumor, and edema/T2-hyperintense tumor areas. Dice coefficients were calculated between those segmentations and the automatic ones.

Finally, we calculated tumor volumes from the segmentation maps and extracted summary statistics from diffusion and perfusion maps in the respective tumor areas.

### 2.4. Statistical Analysis

In a first step, the median values of CBV, ADC, and FA of the whole tumor volume were compared between all of the 18 included WHO 2021 CNS tumor diagnoses.

Subsequently, we compared mean and median values, as well as the 5th and 95th percentiles of CBV, ADC, and FA, between the three main subgroups of adult-type diffuse gliomas, namely, glioblastoma (*IDH* wild type), astrocytoma (*IDH* mutant, 1p/19q non-codeleted), and oligodendroglioma (*IDH* mutant, 1p/19q codeleted). This was done for whole tumor, contrast-enhancing tumor, and edema/T2-hyperintense tumor areas, respectively.

In both cases, statistical significance was tested by applying a one-way ANOVA. Levene’s test for homogeneity of variances was conducted beforehand.

In a separate analysis, we particularly addressed the three histologically different species of WHO grade 4 gliomas, namely, “classical” glioblastoma, molecular glioblastoma (which does not show histological criteria for glioblastoma but exhibits the defining molecular pattern), and astrocytoma WHO grade 4 (formerly referred to as “secondary glioblastoma”). Here, we also compared the above-named values of CBV, ADC, and FA. Again, one-way ANOVA testing was performed to test for statistical significance.

To see which groups differ significantly from each other (in case ANOVA revealed any significant difference), a Tukey Honestly Significant Difference (HSD) post-hoc test was conducted.

All statistical analyses were done in Python 3 using the open-source libraries matplotlib, scipy, and seaborn.

## 3. Results

### 3.1. Characteristics of the Study Population

The study was scheduled from 2020 to 2022, and the patient population consisted of 90 women and 136 men. The patients were 56 years old on average. The different tumor entities with their incidences and gender distribution are shown in Table 1.

**Figure 1:**
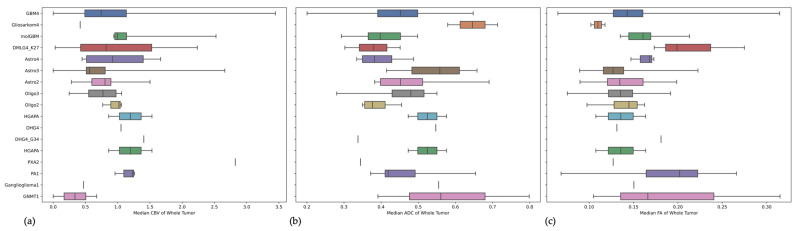
Boxplots illustrating the median values of cerebral blood volume (CBV) (**a**), apparent diffusion coefficient (ADC) from diffusion-weighted imaging (**b**), and fractional anisotropy (FA) from diffusion-tensor imaging (**c**) of the whole tumor volume of all integrated diagnoses as explained in Table 1.

As a first overview, Figure 1 depicts the distribution of median CBV, ADC, and FA values for all 18 integrated diagnoses with respect to the whole tumor volume. While several trends (such as increasing CBV/decreasing ADC) are clearly visible with increasing tumor grade and malignancy, also a large heterogeneity in these imaging metrics can be observed.

A comparison between fully-automated tumor segmentations and manually drawn delineations in a subset of n = 22 glioma patients resulted in median dice scores of 0.86 for whole tumor volume, 0.76 for edema/T2-hyperintense tumor, 0.82 for contrast-enhancing tumor, and 0.79 for necrotic tumor core, respectively. All detailed results can be found in the supplements.

### 3.2. Comparison of Imaging Metrics between the Three Types of Adult-Type Diffuse Gliomas

In a first analysis, CBV, ADC, and FA values were compared between whole tumor (WT), edema/T2-hyperintense tumor, and contrast-enhancing tumor (CET) of the three most common tumor types of gliomas, regardless of tumor grade. Those are glioblastoma (*IDH* wild type), astrocytoma (*IDH* mutant, 1p/19q non-codeleted), and oligodendroglioma (*IDH* mutant, 1p/19q codeleted).

**Figure 2:**
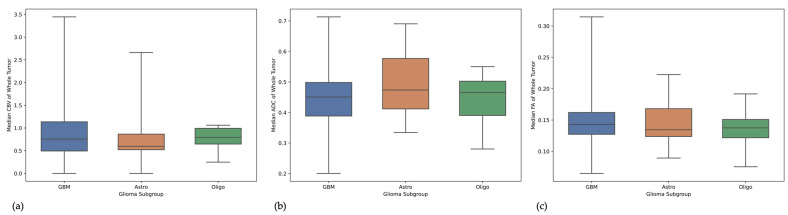
Comparison of median cerebral blood volume (CBV) (**a**), apparent diffusion coefficient (ADC) (**b**), and fractional anisotropy (FA) (**c**) for the three types of adult-type diffuse gliomas, namely glioblastoma (GBM), astrocytoma (Astro), and oligodendroglioma (Oligo).

Here, one-way ANOVA analysis did reveal a significant difference between the median ADC of those entities looking at the whole tumor volume (*p* = 0.0502; Figure 2). More precisely, median ADC was lower in glioblastomas than in astrocytomas (ADC_median_ = 0.448882 vs. ADC_median_ = 0.497266, *p* = 0.0426), while differences between glioblastomas and oligodendrogliomas on the one hand and astrocytomas and oligodendrogliomas on the other hand were not significant (*p* = 0.9884 and *p* = 0.1591, respectively, Tukey HSD post-hoc test).

Further, the median CBV of CET differed significantly between those three entities (*p* = 0.00003). This was due to the much higher median CBV in CET of glioblastoma (CBV_median_ = 2.512548) in comparison to astrocytoma (CBV_median_ = 1.486389) and oligodendroglioma (CBV_median_ = 0.922407).

Looking at the whole tumor volume, a significant difference could only be found in the 95th percentile of CBV (*p* < 0.01), which was again due to higher values in glioblastoma (CBV_p95_ = 3.862419) as opposed to astrocytoma (CBV_p95_ = 2.554698) and oligodendroglioma (CBV_p95_ = 2.688508).

Apart from that, no other significant differences were found between the imaging metrics of the three main groups. All results of ANOVA testing for this subanalysis can be found in Table 2.

### 3.3. Imaging the Novel Three Different Types of WHO Grade 4 Gliomas

**Figure 3:**
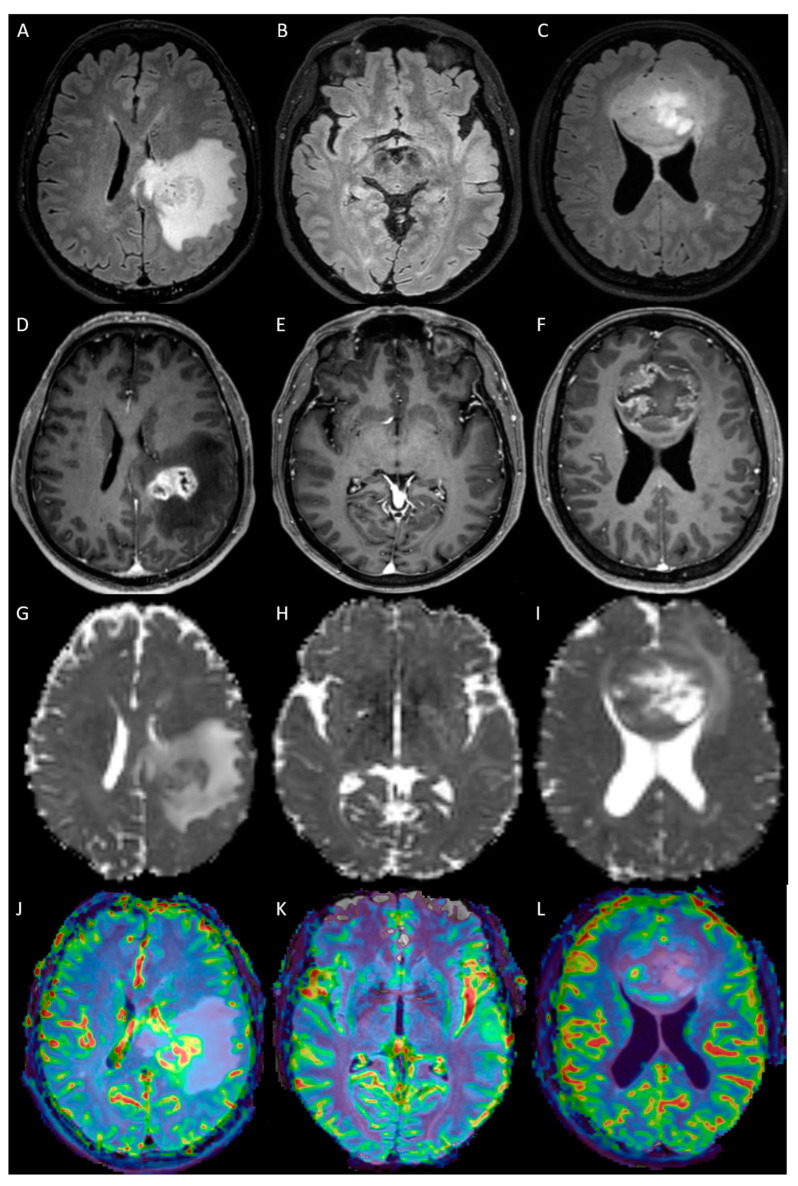
Preoperative MR images of three patients with WHO CNS grade 4 gliomas. The first column shows an example of an astrocytoma of WHO CNS grade 4 (*IDH* mutant). The second column shows images of a patient with only molecularly defined glioblastoma (*IDH* wild type). Note that there is no visible contrast enhancement in the contrast-enhanced T1-weighted image (**D**–**F**). In contrast, the third column depicts a case of “classical” glioblastoma showing typical irregular contrast enhancement surrounding a necrotic tumor core. The sequences depicted are T2-FLAIR (**A**–**C**), contrast-enhanced T1-weighted imaging (**D**–**F**), apparent diffusion coefficient maps (**A**,**C**,**D**,**G**–**I**), and leakage-corrected relative cerebral blood volume maps (rCBV, (**J**–**L**)).

A special focus of this work was laid on the three different tumor types of WHO CNS grade 4 adult-type gliomas as designated by the 5th edition of the WHO classification of tumors of the CNS. Those are conventional glioblastoma (*IDH* wild type) presenting with the classical histological hallmarks of a grade 4 glioma; molecular glioblastoma (*IDH* wild type), harboring any of the glioblastoma-defining genetic alterations, namely TERT promotor mutation, EGFR amplification, or +7/−10 chromosomal copy number changes, without exhibiting the typical histological appearance; and grade 4 astrocytoma (*IDH* mutant, 1p/19q non-codeleted). Figure 3 shows an exemplary patient for each subgroup.

**Figure 4:**
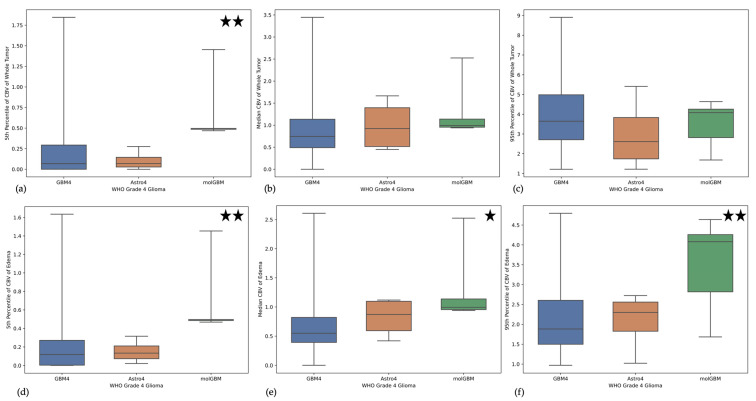
Boxplots illustrating the distribution of 5th percentile, median, and 95th percentile cerebral blood volume (CBV) values in the whole tumor volume (**a**–**c**) and the non-enhancing/edema part (**d**–**f**) for “classical” glioblastoma (GBM4), astrocytoma of WHO CNS grade 4 (Astro 4), and molecular glioblastoma (molGBM). Stars indicate significance of *p* < 0.05 (one star) and *p* < 0.01 (two stars), respectively.

Interestingly, none of the molecular glioblastomas in our cohort showed contrast enhancement. Nevertheless, assessing the whole tumor volume, the median CBV was higher in molecular glioblastomas (CBV_median_ = 1.310136) than in classical glioblastomas (CBV_median_ = 0.968771) and astrocytomas (CBV_median_ = 0.989630), although this difference was not significant (*p* = 0.58685, Figure 4). However, in the 5th percentile of CBV values, this difference was significant, showing much higher values for molecular glioblastoma (CBV_p5_ = 0.679714) than for the other entities (CBV_p5_ = 0.190552, and CBV_p5_ = 0.103663, respectively, *p* = 0.00220, Figure 4).

On the other hand, only addressing the non-enhancing tumor part, the CBV values of molecular glioblastoma were significantly higher for the 5th percentile, median, and 95th percentile (*p* = 0.00111, *p* = 0.01309, and *p* = 0.00295 respectively).

Moreover, looking at the highest values of ADC (ADC_p95_), those were significantly lower in molecular glioblastoma for the whole tumor volume (*p* = 0.02810) and for only the non-enhancing tumor area (*p* = 0.03295), compared to classical glioblastoma and astrocytoma grade 4.

FA values did not show any significant differences between those three groups.

All ANOVA results can be found in Table 3.

## 4. Discussion

With the introduction of the new WHO classification for CNS tumors in 2021, new tumor entities/subclasses have been created by focusing increasingly on molecular characteristics [1]. So far, the interpretation of radiological tumor grading has been mainly based on histological data [2]. Although several studies have already shown a correlation between molecular image markers such as CBV, ADC, or ITSS (intratumoral susceptibility signals, indicating neo-angiogenesis) and tumor type and grade [6,22,23], these data are based on the old WHO classification of 2016 [24]. Only a few groups elaborated on differences based on the novel WHO classification of 2021. Thus, Yang et al. reported differences in ADC values, rCBV, and ITSS in astrocytomas of WHO CNS grades 2, 3, and 4 based on the WHO classification 2021 [3]. However, the CDKN2A/B mutation was not considered. Furthermore, with recently published results, Ma et al. postulate that differentiation of *IDH* status is possible by analysis of ADC parameters. Thus, *IDH* mutant gliomas showed higher ADC values than *IDH* wild-type gliomas. Among *IDH* mutant gliomas, 1p/19q intact gliomas seem to exhibit higher ADC values than 1p/19q codeleted gliomas. In contrast to our study, this analysis was not based on a fully automated extraction of imaging metrics from all tumor volumes but on individually placed regions of interest, which poses a possible source of error [25].

A reevaluation of the available data as well as an analysis of new data is essential for a radiological diagnosis with impact on subsequent therapy planning and prognosis determination in clinical patient care. The aim of this study was to provide an overview of differences in ADC, FA, and rCBV in the different tumor areas (CET, T2-hyperintense tumor area, and WT) between 18 different glioma entities using a retrospective analysis of a mono-centric, prospective cohort of newly diagnosed glioma patients (n = 226). In particular, the WHO CNS grade 4 tumor entities glioblastoma (*IDH* wild type), astrocytoma (*IDH* mutant), and molecularly defined glioblastoma (*IDH* wild type), which have molecular characteristics but lack the histological hallmarks of glioblastoma, should be emphasized. As indicated in many previous studies, the results of this work suggest that ADC and rCBV still reflect the tumor biology and malignancy of tumors well under consideration of the new WHO classification 2021 and thus can be used in the future as a diagnostic tool for specifying entities in more detail. This also creates the basis for future radio-genomics studies assessing the interplay of the tumor genotype and imaging phenotype.

In contrast to previous studies, our work did not find significant differences in FA values between tumor entities [26], which, however, could be explained by excessively high free water content and may need to be corrected by elimination of the free water signal component in the course, which already showed promising results by using a deep-learning-based approach to free water correction (FWC), according to Metz et al. [27].

It will be exciting to see if further analyses will enable the molecular characteristics to be determined on the basis of imaging biomarkers and, consequently, a definite radiological diagnosis to be made even preoperatively.

Of special interest are the differences between the in part newly established WHO CNS grade 4 tumor entities, glioblastoma (*IDH* wild type), astrocytoma (*IDH* mutant) and diffuse glioma (*IDH* wild type) with molecular characteristics of a glioblastoma. Here, the seemingly (in terms of histopathology and T1 contrast enhancement) lower-grade gliomas with molecular characteristics of a glioblastoma (now also defined as a glioblastoma) showed a significantly higher CBV, especially in the T2-hyperintense area, compared to glioblastomas (*IDH* wild type) and astrocytomas (*IDH* mutant). In correlation, there was a significantly lower range of ADC values, indicating a significantly higher proportion of cell-dense tumor areas with less edema in the T2 hyperintense area. These results highlight the importance of advanced imaging and molecular characterization of these tumors, where the biological malignancy of these neoplasms can be seen.

Although our study provides promising results, some limitations still remain. Despite being a large cohort (n = 226) with high heterogeneity of new gliomas according to the new WHO CNS classification 2021, the number of entities diverged significantly, which, however, is also due to the different prevalences.

In addition, there is a limited significance between the image characteristics in the comparison of the three main tumor groups, glioblastoma, oligodendroglioma, and astrocytoma, because different WHO CNS grades are subsumed here, for which distinct imaging and biological differences are already known from previous studies.

## 5. Conclusions

By using AI-based image processing, we found significant differences in tumor biology-associated imaging phenotypes in a diverse cohort of newly-diagnosed gliomas. This work suggests the possibility of preoperative tumor grading by using advanced imaging, resulting in earlier diagnosis and improved individual treatment decisions, and offers exciting avenues towards unraveling genotype—imaging phenotype associations.

## Figures and Tables

**Table 1:** Summary of all 18 included WHO CNS 2021 tumor entities by gender.

Entity	Male	Female	Total
Glioblastomas (*IDH* wild type) WHO CNS grade 4	91	56	147
Oligodenrogliomas (*IDH* mutant, 1p/19q codeleted) WHO CNS grade 3	10	6	16
Oligodendrogliomas (*IDH* mutant, 1p/19q codeleted) WHO CNS grade 2	1	4	5
Astrocytomas *(IDH* mutant) WHO CNS grade 4	1	3	4
Astrocytomas (*IDH* mutant) WHO CNS grade 3 (Astro3)	7	4	11
Astrocytomas (*IDH* mutant) WHO CNS grade 2 (Astro2)	4	5	9
Pilocytic astrocytomas WHO CNS grade 1	6	3	9
Diffuse gliomas (*IDH* wild type) with molecular characteristics of glioblastoma WHO CNS grade 4	6	0	6
Gliosarcomas (*IDH* wild type) WHO CNS grade 4	1	2	3
Glioneural mixed tumors WHO CNS grade 1	1	2	3
Diffuse midline gliomas (H3 K27M mutant) WHO CNS grade 4	3	0	3
High grade astrocytomas with piloid features	0	2	2
*IDH* mutant gliomas WHO CNS grade 2	1	2	3
Diffuse high grade pediatric type glioma WHO CNS grade 4	1	0	1
Diffuse hemispheric glioma; H3 G34- mutant WHO CNS grade 4	1	0	1
Ganglioglioma WHO CNS grade 1	0	1	1
Higher grade glioma; *IDH* wild type	1	1	1
Pleomorphic xanthoastrocytoma WHO CNS grade 2	1	0	1
All gliomas	136	90	226

**Table 2:** Results of one-way ANOVA analysis between the 5th percentile, median, and 95th percentile of cerebral blood volume (CBV), apparent diffusion coefficient (ADC), and fractional anisotropy (FA) of glioblastoma, astrocytoma, and oligodendroglioma. Differences were assessed for the whole tumor volume, non-enhancing tumor/edema, and contrast-enhancing tumor (CET), respectively.

		CBV			ADC			FA		
		5th Percentile	Median	95th Percentile	5th Percentile	Median	95th Percentile	5th Percentile	Median	95th Percentile
**Whole Tumor**	***p*-value ANOVA**	0.70068	0.37781	**<0.01**	0.15987	**0.05027**	0.41801	0.64256	0.28855	0.38134
	***p*** **-value** **Levine**	0.70946	0.15586	0.05249	0.57551	0.31109	0.80807	0.11212	0.83200	0.59977
**Edema/non-enhancing tumor**	***p*-value ANOVA**	0.98535	0.56095	0.38532	0.70645	0.51986	0.45611	0.01222	0.12756	0.82143
	***p*** **-value** **Levine**	0.98536	0.51305	0.53941	0.86890	0.18209	0.90134	0.37560	0.69175	0.72108
**CET**	***p*-value ANOVA**	0.03270	**0.00003**	0.00026	0.15085	0.41333	0.39038	0.81616	0.55772	0.24551
	***p*** **-value** **Levine**	**0.01596**	0.12627	0.43610	0.04164	0.87101	**0.01402**	0.43759	0.65382	0.06869

**Table 3:** Results of one-way ANOVA analysis between the 5th percentile, median, and 95th percentile of cerebral blood volume (CBV), apparent diffusion coefficient (ADC), and fractional anisotropy (FA) of the three different types of WHO CNS grade 4 gliomas, namely “classical” glioblastoma (*IDH* wild type), molecularly defined glioblastoma (*IDH* wild type), and astrocytoma WHO grade 4 (*IDH* mutant). Differences were assessed for the whole tumor volume and the non-enhancing tumor/edema.

		CBV			ADC			FA		
		5th Percentile	Median	95th Percentile	5th Percentile	Median	95th Percentile	5th Percentile	Median	95th Percentile
**Whole Tumor**	*p*-value ANOVA	**0.00220**	0.58685	0.40175	0.34625	0.21793	**0.02810**	0.00994	0.61366	0.57205
	*p*-value Levine	0.76759	0.87670	0.68798	0.85383	0.76440	0.46239	0.39268	0.55937	0.52161
**Edema/non-enhancing tumor**	*p*-value ANOVA	**0.00111**	**0.01309**	**0.00295**	0.28981	0.079690	**0.03295**	0.81053	0.86418	0.27737
	*p*-value Levine	0.77104	0.95884	0.64370	0.94306	0.65422	0.28295	0.36378	0.36073	0.56071

## Data Availability

The genomic and imaging data are not available due to privacy restrictions.

## Supplementary Materials

The following supporting information can be downloaded at: https://www.mdpi.com/article/10.3390/cancers15082355/s1, File S1: Dice Score.
