# Supplementary material for: Imaging the WHO 2021 Brain Tumor Classification: Fully Automated Analysis of Imaging Features of Newly Diagnosed Gliomas
Source: Cancers (Basel). 2023 Apr 18;15(8):2355. doi: 10.3390/cancers15082355 (PMC10136825; doi:10.3390/cancers15082355)
Supplement: Supplementary file 1 [file cancers-15-02355-s001.zip › cancers-2205596-supplementary.pdf]

Dice_Score

| Patient | Necrosis | Non Enhancing Tumor | Contrast Enhancing Tumor | Whole Tumor |
|---|---|---|---|---|
| **preop 031** | 0.943480581876418 | 0.7871588756336081 | 0.7662575439072354 | 0.9155297740537831 |
| **preop 119** | 0.9121043730369655 | 0.758697078782959 | 0.7998235590657032 | 0.8358334494101511 |
| **preop 127** | 0.7904054825813821 | 0.6627300613496933 | 0.837079334224274 | 0.7390737648160971 |
| **preop 102** | 0.7132231404958678 | 0.9122728718681601 | 0.8612707902199739 | 0.9313152807044417 |
| **preop 133** | 0.7827808015833746 | 0.5782688766114181 | 0.7835529979580471 | 0.8289174610713438 |
| **preop 070** | | 0.7466241360978203 | | 0.7466241360978203 |
| **preop 048** | 0.8941952041869008 | 0.7766053092320069 | 0.7105835383024988 | 0.8714004877177002 |
| **preop 083** | 0.8424322064269502 | 0.7126080922387104 | 0.8368336025848142 | 0.852810357735273 |
| **preop 208** | 0.09517241379310348 | 0.803547982376798 | 0.8884737031748362 | 0.8655687445324601 |
| **preop 237** | 0.734955185659411 | 0.8110455462742994 | 0.8632299790755183 | 0.8409082219539772 |
| **preop 051** | 0.8487723214285714 | 0.7663148978223273 | 0.8350827254936843 | 0.8017792351717752 |
| **preop 060** | 0.7350714886459209 | 0.42588163140141067 | 0.8551228393748078 | 0.8696775709167792 |
| **preop 094** | 0.09900990099009899 | 0.809779276517474 | 0.8190874566569432 | 0.8956894358894085 |
| **preop 058** | 0.919165201959011 | 0.6362710878417684 | 0.8126741790863928 | 0.9280136849944332 |
| **preop 032** | 0.9046452387519672 | 0.8157442638805819 | 0.7936077907338593 | 0.9022763180993622 |
| **preop 050** | 0.6051032806804375 | 0.9140081954773107 | 0.803088803088803 | 0.9258456739859503 |
| **preop 125** | | 0.7979197622585439 | | 0.7979197622585439 |
| **preop 114** | | 0.9092282512904444 | | 0.9092282512904444 |
| **preop 112** | 0.0 | 0.5922055569215638 | | 0.7689440332286064 |
| **preop 123** | 0.8997208579202148 | 0.6001692084083563 | 0.7741846541673232 | 0.8634631047401857 |
| **preop 089** | 0.7196819085487077 | 0.75761102025412 | 0.8676804513864997 | 0.8880535223396151 |
| **preop 027** | 0.6847136273864385 | 0.7206262261328982 | 0.7956528218826308 | 0.804926031209255 |
| **Mean** | 0.7291462897750968 | 0.740696282212376 | 0.8168492650213248 | 0.853809013737155 |
| **Median** | 0.7865931420823784 | 0.7625059883026432 | 0.815880817871668 | 0.864515924636323 |
